# Comparative Analysis of Physiological Vergence Angle Calculations from Objective Measurements of Gaze Position

**DOI:** 10.3390/s24248198

**Published:** 2024-12-22

**Authors:** Linda Krauze, Karola Panke, Gunta Krumina, Tatjana Pladere

**Affiliations:** Department of Optometry and Vision Science, Faculty of Science and Technology, University of Latvia, Jelgavas Street 1, LV-1004 Riga, Latvia; gunta.krumina@lu.lv (G.K.); tatjana.pladere@lu.lv (T.P.)

**Keywords:** eccentric photorefractometry, PowerRef 3, kappa angle, calibration, objective vergence

## Abstract

Eccentric photorefractometry is widely used to measure eye refraction, accommodation, gaze position, and pupil size. While the individual calibration of refraction and accommodation data has been extensively studied, gaze measurements have received less attention. PowerRef 3 does not incorporate individual calibration for gaze measurements, resulting in a divergent offset between the measured and expected gaze positions. To address this, we proposed two methods to calculate the physiological vergence angle based on the visual vergence data obtained from PowerRef 3. Twenty-three participants aged 25 ± 4 years viewed Maltese cross stimuli at distances of 25, 30, 50, 70, and 600 cm. The expected vergence angles were calculated considering the individual interpupillary distance at far. Our results demonstrate that the PowerRef 3 gaze data deviated from the expected vergence angles by 9.64 ± 2.73° at 25 cm and 9.25 ± 3.52° at 6 m. The kappa angle calibration method reduced the discrepancy to 3.93 ± 1.19° at 25 cm and 3.70 ± 0.36° at 600 cm, whereas the linear regression method further improved the accuracy to 3.30 ± 0.86° at 25 cm and 0.26 ± 0.01° at 600 cm. Both methods improved the gaze results, with the linear regression calibration method showing greater overall accuracy.

## 1. Introduction

Video-based eye trackers determine gaze direction and ocular movements with high precision by measuring the relative position of the corneal reflection in relation to the pupil center [1]. Available in both screen-based and head-mounted configurations, these eye trackers enable a wide range of experimental applications, such as in visual attention studies [2], cognitive load assessment [3], and applications in virtual and augmented reality [4,5]. The leading eye-tracking systems operate at high frequencies—up to 1000 Hz—making them the preferred choice for detailed eye movement analysis [6]. With technological advancements, there is a growing interest in supplementing vergence measurements with simultaneous measurements of eye accommodation. Real-time data from these combined measurements can provide valuable insights into how visual processes adapt to different viewing conditions with various displays, as highlighted in studies investigating eye movements in headset-based digital environments [4,7,8].

The video-based eccentric photorefractometry device PowerRef 3 (PlusOptix, Nuremberg, Germany), primarily designed to study dynamic eye accommodation, operates at a 50 Hz sampling rate and can be used for dynamic, simultaneous, non-invasive, and repeatable objective measurements of eye refraction, accommodation, gaze position, and pupil size, allowing the assessment of the near reflex triad. While a 50 Hz sampling rate does not sufficiently fulfill the required speed for detailed eye movement studies [9], it remains the most promising commercially available device for researchers interested in real-time, simultaneous accommodation and vergence responses. Common vision research applications that employ PowerRef 3 include exploring infant visual system development [10], investigating accommodation and binocular function disorders [11,12,13], and assessing the effect of novel display technologies on the visual system [14,15].

The accuracy of the measured refraction and accommodation response in devices using eccentric photorefractometry depends on the calibration of the luminance slope formed across the pupil and the defocus calibration factor used to convert the luminance slope into diopters. The PowerRef 3 device uses a universal (population average) defocus calibration factor. Previous studies have highlighted that individual differences in pupil size, refractive error of the eye, and reflectance properties of the retina may, in certain cases, contribute to variations in the individual calibration factor, resulting in deviations from the population average [16,17]. Sravani et al. [18] demonstrated that the accuracy of refraction measurements using photorefraction with a universal calibration factor varies significantly across people with different ethnic origins, suggesting that individual defocus calibration factors are crucial for accurate refraction estimation. The calibration of refraction and accommodation data to obtain an individual defocus calibration factor is performed using an infrared filter and trial lenses [16,17,18]. Previous studies have highlighted that the range of lenses used for individual calibration significantly influences the variability of calibration estimates, where protocols incorporating both positive and negative trial lenses provide the best estimate of the calibration factor [16]. Additionally, it has been suggested to scale the output data according to the magnitude of the induced magnification or minification when using positive and negative trial lenses during the calibration process or when performing any experimental design using ophthalmic lenses [19]. While the accuracy of the measured refraction in video-based eccentric photorefractometry devices has been thoroughly described in the scientific literature [16,17,18,19], the accuracy of vergence measurements has received considerably less attention. To determine the gaze position in degrees, PowerRef 3 employs a calculation method that multiplies the decentration distance between the first Purkinje image and the center of the pupil by the Hirschberg ratio. This calculation utilizes a population-averaged Hirschberg ratio of 11.82°/mm [personal communication]. However, using the population-averaged Hirschberg ratio is susceptible to inaccuracies due to the inter-subject variability of the Hirschberg ratio, which can vary between 7 and 16°/mm [20,21]. Ntodie et al. [21] suggested three different techniques for calculating the Hirschberg ratio: eccentric viewing, theoretical, and prism-based techniques. Their results showed that the eccentric viewing and the theoretical techniques have good repeatability, with respective values of ±0.4°/mm and ±0.3°/mm. However, even after applying the individual Hirschberg ratio, vergence data obtained from Purkinje image eye trackers show an offset between the obtained and expected physiological vergence angles. In humans, the pupillary and visual axes do not align, forming an angle known as the kappa angle, which plays an important role when interpreting the obtained vergence angle. Conventional eye trackers employing calibration procedures account for the kappa angle in gaze measurements [22], whereas Purkinje image eye trackers, such as the PowerRef 3, do not incorporate individual calibration for gaze measurements.

Most individuals have a positive kappa angle, where the fovea is positioned temporally relative to the pupillary axis [23]. When the pupillary axes are parallel (as expected during viewing at infinity), the visual axes converge by the magnitude of the positive kappa angle. As a result, for a positive kappa angle, the physiological vergence angle is typically larger than the visual angle observed and measured using first Purkinje image-based eye trackers. The kappa angle varies among individuals, with an average value of approximately 5° [24]. The individual kappa angle is affected by various factors:

- Type of refractive error: In hyperopic eyes, the kappa angle is larger compared to myopic eyes [25].
- Age: Kappa angle decreases by 0.015° per year [26].
- Measurement method: The principle of the measuring device significantly affects the measured kappa angle [24,25,27].
- Eye laterality: Previous studies have observed a larger kappa angle in the left eye [25,28].

Therefore, because devices such as PowerRef 3 lack individual calibration for gaze measurements, previous studies have been limited to assessing relative changes in vergence rather than the absolute vergence status [12,29]. This limitation causes the measured vergence angles to appear divergent from the target, even in binocularly aligned conditions at near distances, where convergence is expected, thereby hindering the evaluation of physiological vergence at the individual level. Moreover, the expected vergence angle, defined as ideal gaze alignment at the corresponding stimulus distance, is rarely achieved physiologically due to inherent visual system limitations, such as vergence error, requiring a more complex correction methodology than simple linear regression.

The absence of a methodology to address this issue has led researchers to focus on assessing relative changes or changes at the group level, hindering the ability to evaluate the physiological vergence angle at the individual level. To date, a universal method for determining physiological vergence angles from the visible vergence angle provided by the first Purkinje image eye trackers has not been described. Recently, Liu et al. [22] suggested the use of an automated algorithm based on the initial assumption of a theoretical kappa angle of 5° with a promising but rather complex algorithm verification procedure that demonstrated a gaze accuracy of 1.30° in the horizontal plane and 1.34° in the vertical plane. Another approach was proposed by Kooijman et al. [29], in which an 11.2° offset to the visual vergence angle at a distance of 1.8 m was used, assuming a population-averaged interpupillary distance of 63 mm; however, this offset value should be modified if a distance other than 1.8 m is used. Thus, the purpose of our study was to propose and compare new approaches for calculating the physiological vergence angle using data obtained with the first Purkinje image-based eye trackers.

## 2. Materials and Methods

### 2.1. Participants

A total of 23 individuals (20 females, 3 males) without binocular vision problems participated in this study (mean age ± SD: 25 ± 4 years). Based on objective refraction obtained at a 6 m distance with PowerRef 3, 70% of participants were emmetropic (ranging from >−0.50 to +0.50 D), while 30% had myopia (≤−0.50 D). Average *IPD* for females was 62.4 ± 2.4 mm (min 59.2 mm, max 66.9 mm), and 65.7 ± 1.9 mm (min 64.0 mm, max 67.7 mm) for males. The study was conducted in accordance with the Declaration of Helsinki and approved by a Research Ethics Committee at the University of Latvia (No. 18-29/38, Date: 3 February 2024).

### 2.2. Gaze and Interpupillary Distance Measurements

Vergence measurements were obtained with an eccentric photorefractometer PowerRef 3 (PlusOptix, Nuremberg, Germany), which allows binocular data to be obtained at a frequency of 50 Hz, with a 0.01 D step for refraction measurements and 0.1 mm for pupil size and first Purkinje image location. All measurements were performed under low room lighting conditions with an illumination range of 60–140 lx. The baseline illumination in the room was set to 140 lx, and the illumination was reduced for individuals whose pupil size was too small to obtain measurements (<4 mm), which, when it occurred, was mostly at viewing distances ≤ 30 cm.

**Figure 1:**
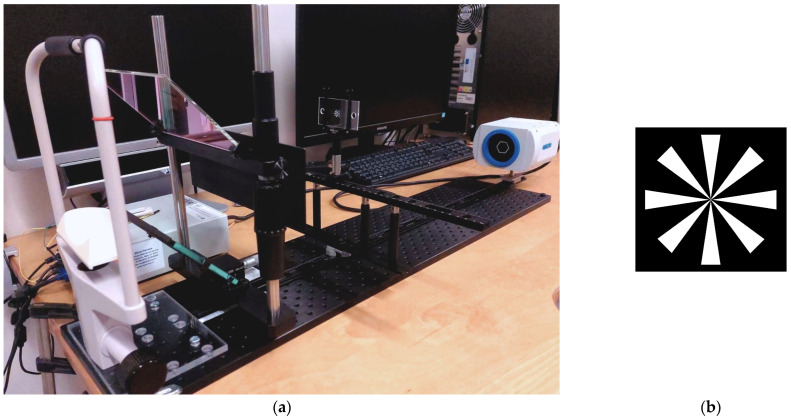
PowerRef 3 setup with custom-built stimulus construction on rails (**a**), and Maltese cross stimulus (**b**).

A forehead and chin rest were employed to minimize head movements and to ensure that the chosen stimulus distance remained well controlled. The Maltese cross stimulus was mounted on a sliding rail construction along the same axis as the chin and forehead rest, ensuring that it remained well centered relative to the midline of the participant’s head while enabling the movement of the stimulus between near distances of 25 cm and 70 cm (Figure 1a). A larger Maltese cross (Figure 1b), with an angular size of 5°, was positioned at 6 m and aligned at the same height and centration. The participants were instructed to look at the middle of the Maltese cross stimulus, blink naturally, and not move their head. Vergence and binocular interpupillary distance measurements were acquired at five distances: 25, 30, 50, 70, and 600 cm. At each distance, data were collected for a period of 10 s, during which participants were instructed to maintain their gaze at the center of the Maltese cross stimulus. Episodes of blinking were excluded from the dataset by considering both data during blinking and recovery from the blink (0.2 s) [30].

Two methods were developed to calculate the total physiological vergence angle. These methods utilize population-averaged values for key anatomical parameters: the center of eye rotation (*COR*) at 15.3 mm [31], the nodal point (*N*) at 7.2 mm [32], and the anterior chamber depth (*ACD*) at 3 mm [33]. Nevertheless, if these parameters can be measured independently, they can be incorporated into the formulas for more precise calculations of the physiological vergence angle.

### 2.3. Physiological Vergence Angle Calculations

To compare the results obtained with the expected physiological vergence angles for each specified distance, the expected vergence angle (*EVA*) was calculated considering the interpupillary distance (*IPD_far_*) at a far distance using the following equation:

(1) $$EVA=2\;\times \;atan\left(\frac{{IPD}_{far}\times 0.5}{d+ACD}\right)$$

where *EVA* is the expected vergence angle (assuming zero fixation disparity) for the specified distance (°), *IPD_far_* is the interpupillary distance at far obtained with PowerRef 3 (m), *d* is the specified distance (m), and *ACD* is anterior chamber depth (assumed 0.003) (m).

To calculate the physiological vergence angle from first Purkinje image-based eye trackers, we are proposing and comparing two methods using data obtained from PowerRef 3. In the kappa angle calibration method, measurement of the kappa angle is required and it was obtained by considering the decentration of the first Purkinje image from the center of the pupil. According to geometric principles, kappa angle was determined as the angle formed at the nodal point between the pupillary and visual axes. In the linear regression method, we used the expected interpupillary distance at a specified near distance (*EIPD*) calculated at the entrance pupil plane and the expected vergence angle (*EVA*) to obtain a linear regression curve that can be further used to estimate the total physiological vergence angle that accounts for the actual interpupillary distance at a specified distance.

#### 2.3.1. Method 1: Kappa Angle Calibration

(2) $$\kappa_{OD}=-atan\left(\frac{{decX}_{OD}}{N}\right)$$

(3) $$\kappa_{OS}=atan\;\left(\frac{{decX}_{OS}}{N}\right)$$

where κ is the kappa angle (with negative values for the negative kappa angle and positive values for the positive kappa angle) (°), *decX* is horizontal decentration from the pupil center at far obtained with the PowerRef 3, *N* is distance from nodal point to cornea apex (assumed 0.0072) (m), is the visual vergence angle obtained with PowerRef 3, OD and OS refer to the measurements for the right and left eyes, respectively, and *TPVA* is the total physiological vergence angle.

(4) $$TPVA=VVA+\kappa_{OD}+\kappa_{OS}$$

#### 2.3.2. Method 2: Linear Regression Calibration

To plot the linear regression curve, the results of *EIPD* (see Equation (5)) and *EVA* (see Equation (1)) should be calculated for at least two specified distances, with one distance at a far point. The *EIPD* values should be plotted on the Y-axis, while *EVA* values should be plotted on the X-axis.

(5) $$EIPD={IPD}_{far}-\left(tan\left(atan\left(\frac{{IPD}_{far}\times 0.5}{d+COR}\right)\right)\times (COR-ACD)\times 2\right)$$

where *EIPD* is expected interpupillary distance at specified distance (m), *IPD_far_* is the interpupillary distance at far obtained with PowerRef 3 (m), *d* is the specified distance (m), *COR* is the center of eye rotation (assumed 0.0152) (m), and *ACD* is anterior chamber depth (assumed 0.003) (m).

When plotting the linear regression curve from the data of *EIPD* and *EVA*, represented by the equation *y* = *mx* + *b*, the slope (*m*) and intercept (*b*) values can be extracted and used in further calculations. These values are necessary for determining the total physiological vergence angle, as described in Equation (6). This calculation incorporates the actual interpupillary distance measured at a specified distance using the PowerRef 3. The slope represents the rate of change in vergence angle relative to changes in the interpupillary distance at a specified distance, while the intercept defines a starting point for the *IPD* at specified distance, if the *EVA* is set to zero.

(6) $$TPVA=\frac{{IPD}_{d}-b}{m}$$

where *TPVA* is the total physiological vergence angle (°), *IPD_d_* is the interpupillary distance obtained with PowerRef 3 at a specified distance (m), *b* is the intercept value, and *m* is the slope value.

### 2.4. Statistical Analysis

Statistical analysis was performed using SPSS (version 22.0; IBM Corporation, Armonk, NY, USA), and visualizations were performed in Microsoft Excel (Microsoft 365). The Shapiro–Wilk test was used to assess the normality of the data distributions. A paired-sample *t*-test was performed to compare the kappa angle between the right and left eyes. A repeated-measures ANOVA was conducted to evaluate the effect of the vergence angle differences at 5 distances and to compare the vergence angle results yielded by the kappa and linear regression methods. Mauchly’s test of sphericity was used to test whether the assumption of sphericity was met in a repeated-measures ANOVA. Greenhouse–Geisser’s correction was reported whenever Mauchly’s test of sphericity was violated. Statistical significance was set at *p* < 0.05.

## 3. Results

Our results demonstrate that the visual vergence data obtained with PowerRef 3 measured significantly more divergent gaze positions than the expected vergence angle at distances from 25 cm to 6 m (see Table 1). The average difference between visual vergence data and the expected vergence angle at each distance was as follows: 9.64 ± 2.73° at 25 cm distance with a 95% confidence interval of 8.45° to 10.82° (t(22) = 16.91, *p* < 0.01); 9.81 ± 2.63° at 30 cm distance with a 95% confidence interval of 8.68° to 10.95° (t(22) = 17.91 *p* < 0.01); 9.87 ± 2.60° at 50 cm distance with a 95% confidence interval of 8.74° to 10.99° (t(22) = 18.20, *p* < 0.01); 9.49 ± 2.80° at 70 cm distance with a 95% confidence interval of 8.28° to 10.70° (t(22) = 16.24, *p* < 0.01); and 9.25 ± 3.52° at 6 m distance with a 95% confidence interval of 7.72° to 10.77° (t(22) = 12.60, *p* < 0.01). This discrepancy significantly differs from the expected physiological vergence angle and highlights the importance of performing additional calculations using visual data to accurately interpret the vergence results obtained with the first Purkinje image-based eye trackers.

The average kappa angle of the right eye (0.32 ± 0.16 mm or 2.51 ± 1.29°) was significantly smaller than the average kappa angle of the left eye (0.42 ± 0.16 mm or 3.31 ± 1.31°) (t(22) = −3.48, *p* < 0.01). The average difference in kappa angle between the right and left eye was −0.80 ± 1.10°, with a 95% confidence interval of −1.28° to −0.32°.

At all distances, both methods yielded results that were significantly different from the expected vergence angle: at 25 cm (F(94.75, 2) = 103.65, *p* < 0.01), at 30 cm (F(94.69, 2) = 89.94, *p* < 0.01), at 50 cm (F(140.31, 2) = 90.12, *p* < 0.01), at 70 cm (F(105.35, 2) = 76.17, *p* < 0.01), and at 600 cm (F(100.13, 2) = 97.88, *p* < 0.01). The amount by which the kappa angle results deviate from the *EVA* remains relatively consistent across various distances, whereas, for the linear regression method, the difference is distance-dependent. For the kappa calibration method, the mean difference between the *EVA* was 3.96 ± 0.31° at 25 cm distance, 3.84 ± 0.29° at 30 cm, 3.96 ± 0.21° at 50 cm, 3.62 ± 0.24° at 70 cm, and 3.70 ± 0.36° at 600 cm. For the linear regression method, the mean difference between the *EVA* progressively decreases with increasing distance: 3.30 ± 0.25° at 25 cm distance, 2.75 ± 0.23° at 30 cm, 2.00 ± 0.20° at 50 cm, 1.46 ± 0.19° at 70 cm, and 0.26 ± 0.01° at 600 cm.

To compare the results of the total physiological vergence angle obtained with the kappa and linear regression methods at all distances, repeated measures ANOVA was conducted. Mauchly’s test of sphericity indicated that the assumption of sphericity was violated for the interaction effect of distance χ^2^(9) = 128.16, *p* < 0.01. Therefore, the degrees of freedom were corrected using the Greenhouse–Geisser estimate of sphericity (ε = 0.46). The main effect of distance was significant, F(52.99, 1.86) = 28.54, *p* < 0.01, indicating that the physiological vergence angles varied between distances, regardless of the method, as expected. At the closest distance (25 cm), both methods provided similar results (F(3.43, 1) = 5.07, *p* = 0.07), while for farther distances, there was a statistically significant difference between the two methods, where the total physiological angle with both methods was smaller (more divergent) than the *EVA*, and the linear regression method gave a slightly larger (more convergent) angle compared to the kappa angle method; therefore, the angle was closer to the expected vergence angle. At a 30 cm distance, the difference between both methods was −1.09 ± 1.09° (F(10.35, 1) = 13.55, *p* < 0.01), 50 cm −1.96 ± 1.01° (F(48.10, 1) = 44.30, *p* < 0.01), 70 cm −2.16 ± 1.01° (F(50.35, 1) = 53.51, *p* < 0.01), and 600 cm −3.43 ± 1.72° (F(92.54, 1) = 135.66, *p* < 0.01).

To determine whether the difference between the expected vergence angle and the total physiological vergence angle could be attributed to independent variables, we tested the subjective fixation disparity (measured with a Saladin card at a 40 cm distance (Bernell Corporation, Mishawaka, Indiana, USA)) by plotting individual differences at measured distances (25 cm, 30 cm, 50 cm, and 70 cm) and extrapolating the difference at 40 cm using a regression slope, as this distance was not directly measured, while also considering the potential influence of the kappa angle. For the linear regression calibration method, the ANOVA test showed no significant effect of the independent variables on the difference from the *EVA* (F(2, 13) = 1.04, *p* = 0.38), with neither the fixation disparity (t = 1.32, *p* = 0.21) nor the kappa angle (t = −0.58, *p* = 0.57) being significant contributors. For the kappa calibration method, the fixation disparity was used as a variable and showed no significant effect (F(1, 14) = 3.38, *p* = 0.09).

**Figure 2:**
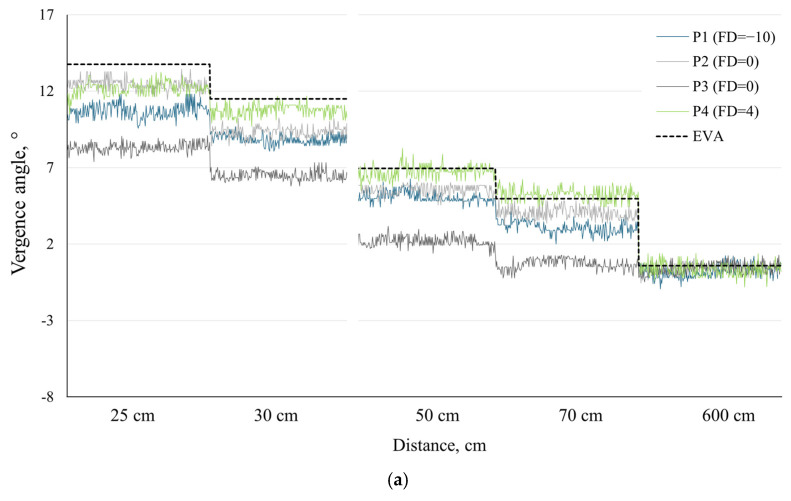

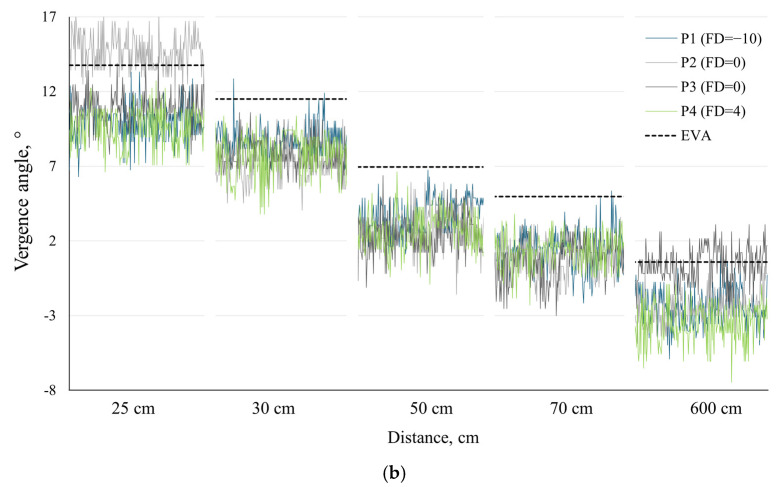
Expected vergence angle (*EVA*, black dashed line) and total physiological vergence angle obtained using the two proposed calibration methods: (**a**) kappa angle calibration and (**b**) linear regression calibration. Gaze data are represented over a duration of 4 s at each of the 5 distances for four participants: P1 (FD = −10, largest exo fixation disparity, blue line), P2 (FD = 0, closest to *EVA*, light gray line), P3 (FD = 0, largest deviation from *EVA*, dark gray line), and P4 (FD = 4, largest eso fixation disparity, green line). FD—fixation disparity.

To illustrate and demonstrate the potential applications of our proposed methods, we chose to present individual data for four participants from our sample: P1 and P4, who had the highest exo and eso fixation disparities, respectively, and two participants both with zero fixation disparity—one exhibiting the largest and the other the smallest deviations from the expected vergence angle (*EVA*) among our participants (see Figure 2). Figure 2a,b present the *TPVA* results for each participant using the linear regression method and kappa angle calibration method, respectively, at five different distances.

## 4. Discussion

The simultaneous evaluation of dynamic accommodation and vergence responses, permitted by eccentric photorefractometry, is of high interest in vision research, offering valuable insights into real-time ocular behavior under diverse experimental conditions and designs. Our study has demonstrated that calibration calculations are essential for determining the physiological vergence angle from PowerRef 3 measurements, as the recorded visual vergence data output by the first Purkinje image-based eye-tracking principle provides a visual vergence angle that is substantially more divergent than the expected values at corresponding distances.

Both proposed methods are influenced by different parameters. For the kappa angle calibration method, the primary influencing factors are the decentration value obtained with the PowerRef 3 and the assumed location of the nodal point. Given the substantial variability in the results produced by different devices measuring the kappa angle [25,27], one of the limitations of the kappa angle calibration method is that it is directly influenced by the specific technique used for kappa angle measurement. For example, Domínguez-Vicent et al. [27] evaluated the difference in kappa angle results between Orbscan II and Galilei G4 devices and found that the kappa angle difference was 0.16 ± 0.08 mm. A change of ±0.16 mm in horizontal decentration measured with PowerRef3 would lead to a corresponding 1.27 ± 0.003° change in the kappa angle of each eye, which would result in a total change of 2.54 ± 0.01° in the total physiological vergence angle (*TPVA*). Since significant differences (up to 3.6°) [27] in the kappa angle can be caused by the differences between measuring devices, and to our knowledge there is no previous research comparing the kappa angle results obtained from PowerRef 3 and other devices, this may explain the discrepancy between the kappa angle calibration method and the expected vergence angle (*EVA*), as the obtained result differences ranged from 3.62 to 3.96°. For kappa angle measurements derived from the distance between the first Purkinje image and the pupil center, high repeatability is expected (SD = 0.04 mm over 10 trials) [34]. However, when using devices with different measurement principles, it is advisable to take multiple kappa angle measurements and use their average to improve accuracy [24,26]. The calculations using the kappa angle calibration method are influenced by how the stimulus is centered relative to the participant, which was ensured by using a sliding rail aligned with the midline of the participant’s head. Additionally, the kappa angle value calculated at a far distance was applied to the total physiological vergence angle (*TPVA*) calculation at near distances, as recent studies have shown no significant variation in kappa angle across varying distances [27]. Consequently, this could clarify why the differences between the *TPVA* and *EVA* results remained constant across different distances. Stability in the stimulus setup, including a forehead rest, is critical, especially at closer distances where small shifts (e.g., 1 cm at 25 cm) can alter the vergence angle by ~0.5°.

In the linear regression method, the results are affected by the individual *ACD*, whereby an increase in the *ACD* results in a reduction in the expected vergence angle, with a decrease of approximately 0.03° for every 0.5 mm increase in the *ACD* at a 25 cm distance. The *ACD* affected the results less at distances beyond 25 cm, but the *TPVA* calculated with the linear regression method increases with a larger *ACD*. Therefore, if the *ACD* of the participant is larger than the assumed value of 3 mm, the resulting vergence angle is expected to be closer to the *EVA*. Conversely, if the *ACD* is less than 3 mm, the vergence angle will deviate further from the *EVA*. Given that *ACD* values vary individually, typically ranging between 2.80 and 3.75 mm [35], when evaluating the difference in *TPVA* at the typical minimum and maximum *ACD* values, the result would be 0.9° closer to the *EVA* when the *ACD* = 3.75 mm compared to the *ACD* = 2.8 mm. It should be noted that the far *IPD* value used for the expected interpupillary distance calculations in the linear regression method is obtained at a far distance using PowerRef 3. Based on this far-distance *IPD*, the expected *IPD* at closer distances in the pupil plane is theoretically calculated. Although *IPD* is a variable in the linear regression method, the calculated *IPD* and the *IPD* obtained from the PowerRef 3 did not differ significantly (F(0.52, 1) = 12.74, *p* = 0.47). Therefore, although *IPD* may not be a primary contributing factor to the differences between the methods, slight variations still exist. Specifically, if the far-distance *IPD* is smaller, the expected *IPD* value difference across various distances changes slightly less, and participants with smaller far *IPD* values tend to have a lower intercept value than those with larger far *IPD* values.

Both methods show a slight underestimation of the vergence angle, which reflects under-convergence, as the *TPVA* is smaller than the *EVA*. A binocular calibration will not detect this physiological condition, as it assumes the absence of a fixation disparity—where the two lines of sight cross precisely on the fixation target [36]. The average objective fixation disparity has been estimated to rarely exceed 30 arcmin, when measured subjectively and converted to degrees that do not exceed 0.5°, and up to 60 arcmin (1°) or more when measured objectively [36,37]. Under high vergence demands or visual strain, vergence errors can reach 2° [38]. However, in this study, the difference between the *TPVA* and expected vergence angles was 3.30 ± 0.86° at 25 cm, 2.75 ± 0.77° at 30 cm, 2.00 ± 0.83° at 50 cm, and 1.46 ± 0.85° at 70 cm using the linear regression method and 3.93 ± 1.19° at 25 cm, 3.84 ± 1.12° at 30 cm, 3.96 ± 0.89° at 50 cm, and 3.62 ± 1.08° at 70 cm using the kappa angle calibration method. While it was anticipated that the obtained total physiological vergence angle (*TPVA*) would not perfectly align with the expected angle due to various physiological factors, it is important to note that the observed difference exceeds what could be attributed to the fixation disparity alone. Examining the results, the kappa angle calibration method induces a divergent eye position (−3.10 ± 1.71°) at a 6 m distance, and the difference between the kappa angle calibration method and the expected vergence angle (*EVA*) remains relatively consistent at closer distances, while also demonstrating greater variability in the *TPVA* data (see Figure 2b) compared to the *TPVA* obtained using the linear regression calibration method (see Figure 2a). This may indicate a consistent underestimation of the kappa angle, which, in this case, was based on the measurements provided by the PowerRef 3. In contrast, with the linear regression method, the difference between the *TPVA* and *EVA* was not constant across all distances—it decreased for farther stimuli. The difference between both methods at the closest distance was small (−0.65 ± 1.18° at 25 cm), while at a distance of 6 m, the difference increased to −3.43 ± 1.72°, making the *TPVA* obtained with the linear regression method closer to the *EVA* and therefore more precise at farther distances.

This study has certain limitations that should be acknowledged. Firstly, although the experimental setup was well centered, it did not fully control minor head movements, as the head of the participant was on a chin and head rest rather than in a fully fixed position. Secondly, the kappa angle was obtained from PowerRef 3 data, and to the best of our knowledge, no study has compared these results with those from more commonly used measurement techniques, thus leaving open the possibility that the kappa angle may have been underestimated. Furthermore, applying a single kappa angle value obtained at a far distance to near-distance calculations may introduce inaccuracies if the kappa angle varies across distances. Thirdly, the individual variability in parameters such as the anterior chamber depth, nodal point, and center of eye rotation were assumed to correspond to the average population values, potentially overlooking minor differences that could affect the *TPVA* calculation. Finally, binocular *IPD* values were utilized, as PowerRef 3 does not provide monocular *IPD* values; consequently, in cases with an asymmetric monocular *IPD*, the influence of asymmetric fixation disparity might be overlooked.

## 5. Conclusions

This study demonstrates that the gaze angle measured by PowerRef 3 significantly deviates from the expected vergence angle. The proposed calibration methods, kappa angle calibration and linear regression calibration, were successfully demonstrated to substantially reduce this discrepancy. At a distance of 25 cm, both methods produced comparable results, whereas at farther distances, the linear regression method provided values closer to the expected vergence angle. Therefore, both methods can be considered equally effective at close distances, with the linear regression method demonstrating significantly closer results at farther distances, making it more effective overall due to its broader applicability across varying distances.

## Figures and Tables

**Table 1:** The visual and expected vergence angles ((avg. ± SD),°) and calculated vergence angles using the proposed methods.

Distance (*d*), cm	Visual Vergence Angle (*VVA*), °			Expected Vergence Angle (*EVA*), °			Method 1: Kappa Angle Calibration			Method 2: Linear Regression Calibration		
	avg. ± SD	95% CI (Lower)	95% CI (Upper)	avg. ± SD	95% CI (Lower)	95% CI (Upper)	avg. ± SD	95% CI (Lower)	95% CI (Upper)	avg. ± SD	95% CI (Lower)	95% CI (Upper)
25	4.38 ± 2.46	3.32	5.45	14.16 ± 0.57	13.92	14.41	10.20 ± 1.36	9.61	10.79	10.87 ± 1.05	10.41	11.32
30	2.15 ± 2.55	1.05	3.25	11.85 ± 0.48	11.64	12.05	8.01 ± 1.29	7.45	8.57	9.09 ± 0.98	8.67	9.52
50	−2.67 ± 2.53	−3.77	−1.58	7.15 ± 0.29	7.03	7.28	3.19 ± 0.99	2.77	3.62	5.16 ± 0.93	4.75	5.56
70	−4.35 ± 2.77	−5.54	−3.15	5.12 ± 0.21	5.03	5.21	1.50 ± 1.14	1.01	2.00	3.66 ± 0.91	3.27	4.05
600	−8.64 ± 3.51	−10.16	−7.13	0.60 ± 0.02	0.59	0.61	−3.10 ± 1.71	−3.84	−2.36	0.34 ± 0.02	0.33	0.35

## Data Availability

Data are available on reasonable request to the corresponding author.

## References

[1] Carter B.T., Luke S.G. (2020). Best practices in eye tracking research. Int. J. Psychophysiol..

[2] Vehlen A., Spenthof I., Tönsing D., Heinrichs M., Domes G. (2021). Evaluation of an eye tracking setup for studying visual attention in face-to-face conversations. Sci. Rep..

[3] Krejtz K., Duchowski A.T., Niedzielska A., Biele C., Krejtz I. (2018). Eye tracking cognitive load using pupil diameter and microsaccades with fixed gaze. PLoS ONE.

[4] Adhanom I.B., MacNeilage P., Folmer E. (2023). Eye tracking in virtual reality: A broad review of applications and challenges. Virtual Real..

[5] Pladere T., Svarverud E., Krumina G., Gilson S.J., Baraas R.C. (2022). Inclusivity in stereoscopic XR: Human vision first. Front. Virtual Real..

[6] González-Vides L., Hernández-Verdejo J.L., Cañadas-Suárez P. (2023). Eye tracking in optometry: A systematic review. J. Eye Mov. Res..

[7] Aizenman A.M., Koulieris G.A., Gibaldi A., Sehgal V., Levi D.M., Banks M.S. (2023). The statistics of eye movements and binocular disparities during VR gaming: Implications for headset design. ACM Trans. Graph..

[8] Kapp S., Barz M., Mukhametov S., Sonntag D., Kuhn J. (2021). ARETT: Augmented reality eye tracking toolkit for head mounted displays. Sensors.

[9] Andersson R., Nyström M., Holmqvist K. (2010). Sampling frequency and eye-tracking measures: How speed affects durations, latencies, and more. J. Eye Mov. Res..

[10] Horwood A.M., Toor S.S., Riddell P.M. (2015). Convergence and accommodation development is preprogrammed in premature infants. Investig. Ophthalmol. Vis. Sci..

[11] Ntodie M., Saunders K., Little J.A. (2023). Accuracy and stability of accommodation and vergence responses during sustained near tasks in uncorrected hyperopes. Sci. Rep..

[12] Mestre C., Neupane S., Manh V., Tarczy-Hornoch K., Candy T.R. (2023). Vergence and accommodation responses in the control of intermittent exotropia. Ophthalmic Physiol. Opt..

[13] Toor S., Horwood A., Riddell P. (2019). The effect of asymmetrical accommodation on anisometropic amblyopia treatment outcomes. J. AAPOS.

[14] Panke K., Pladere T., Velina M., Svede A., Ikaunieks G., Krumina G. Ocular Performance Evaluation—How Prolonged Near Work with Virtual and Real 3D Image Modifies our Visual System. Proceedings of the 2nd International Conference on Applications of Intelligent Systems, APPIS.

[15] Panke K., Pladere T., Velina M., Svede A., Krumina G. (2019). Objective user visual experience evaluation when working with virtual pixel-based 3D system and real voxel-based 3D system. Photonics.

[16] Bharadwaj S.R., Sravani N.G., Little J.A., Narasaiah A., Wong V., Woodburn R., Candy T.R. (2013). Empirical variability in the calibration of slope-based eccentric photorefraction. J. Opt. Soc. Am..

[17] Ghahghaei S., Reed O., Candy T.R., Chandna A. (2019). Calibration of the PlusOptix PowerRef 3 with change in viewing distance, adult age and refractive error. Ophthal. Physiol. Opt..

[18] Sravani N., Nilagiri V., Bharadwaj S. (2015). Photorefraction estimates of refractive power varies with the ethnic origin of human eyes. Sci. Rep..

[19] Bharadwaj S.R., Bandela P.K., Nilagiri V.K. (2018). Lens magnification affects the estimates of refractive error obtained using eccentric infrared photorefraction. J. Opt. Soc. Am..

[20] Jagini K.K., Vaidyanath H., Bharadwaj S.R. (2014). Utility of theoretical Hirschberg ratio for gaze position calibration. Optom. Vis. Sci..

[21] Ntodie M., Bharadwaj S.R., Balaji S., Saunders K.J., Little J.A. (2019). Comparison of three gaze-position calibration techniques in the first Purkinje image-based eye trackers. Optom. Vis. Sci..

[22] Liu J., Chi J., Sun H. (2023). An automatic calibration method for Kappa angle based on a binocular gaze constraint. Sensors.

[23] Linke L., Horstmann G. (2022). How vergence influences the perception of being looked at. Perception.

[24] Awad-Allah M.A.A., Gharieb H.M., Zaki R.G.E., Othman I.S. (2023). Angle Kappa agreement between Scheimpflug tomography, combined placido Scheimpflug and combined slit scanning placido systems. Int. J. Ophthalmol..

[25] Basmak H., Sahin A., Yildirim N., Papakostas T.D., Kanellopoulos A.J. (2007). Measurement of angle kappa with synoptophore and Orbscan II in a normal population. J. Refract. Surg..

[26] Hashemi H., Khabazkhoob M., Yazdani K., Mehravaran S., Jafarzadejpur E., Fotouhi A. (2010). Distribution of angle kappa measurements with Orbscan II in a population-based survey. J. Refract. Surg..

[27] Domínguez-Vicent A., Monsálvez-Romín D., Pérez-Vives C., Ferrer-Blasco T., Montés-Micó R. (2014). Measurement of angle Kappa with Orbscan II and Galilei G4: Effect of accommodation. Graefes Arch. Clin. Exp. Ophthalmol..

[28] Deng W.Q., Fang Y.H., Lin S.H., Li Y.J. (2022). Dynamic distribution and correlation analysis of the angle kappa in myopia patients undergoing femtosecond-assisted laser in situ keratomileusis. Medicine.

[29] Kooijman L., Dodou D., Jansen S.T., Themans T.S., Russell J.N.M., Petermeijer S.M., Doorman J.R.C., Hablé J.H., Neubert D.S., Vos M.J.C. (2021). Is accommodation a confounder in pupillometry research?. Biol. Psychol..

[30] Horwood A.M., Riddell P.M. (2008). The use of cues to convergence and accommodation in naïve, uninstructed participants. Vision Res..

[31] Ohlendorf A., Schaeffel F., Wahl S. (2020). Positions of the horizontal and vertical centre of rotation in eyes with different refractive errors. Ophthalmic. Physiol. Opt..

[32] Greivenkamp J.E. (2004). Field Guide to Geometrical Optics.

[33] Barrett B.T., McGraw P.V. (1998). Clinical assessment of anterior chamber depth. Ophthalmic. Physiol. Opt..

[34] Yang Y., Thompson K., Burns S.A. (2002). Pupil location under mesopic, photopic, and pharmacologically dilated conditions. Investig. Ophthalmol. Vis. Sci..

[35] Niu L., Luo X., Chen X., Wang X., Zhou X., Qian Y. (2023). Anterior segment characteristics of eyes with anterior chamber depth less than 2.8 mm and axial length greater than 25 mm. Ophthalmol. Ther..

[36] Svede A., Treija E., Jaschinski W., Krumina G. (2015). Monocular versus binocular calibrations in evaluating fixation disparity with a video-based eye-tracker. Perception.

[37] Jaschinski W., Jainta S., Kloke W.B. (2010). Objective vs subjective measures of fixation disparity for short and long fixation periods. Ophthalmic. Physiol. Opt..

[38] Jaschinski W. (2018). Individual objective versus subjective fixation disparity as a function of forced vergence. PLoS ONE.

